# Tumor-Associated Antigens (TAAs) for the Serological Diagnosis of Osteosarcoma

**DOI:** 10.3389/fimmu.2021.665106

**Published:** 2021-04-30

**Authors:** Jitian Li, Bo Qin, Manyu Huang, Yan Ma, Dongsheng Li, Wuyin Li, Zhiping Guo

**Affiliations:** ^1^ Henan Luoyang Orthopedic Hospital (Henan Provincial Orthopedic Hospital)/Henan Institute of Orthopedic and Traumatology, Luoyang, China; ^2^ Transitional Medical Center, The First Affiliated Hospital of Zhengzhou University, Zhengzhou, China

**Keywords:** osteosarcoma, tumor-associated antigen, autoantibodies, biomarker, immunodiagnosis, prognosis

## Abstract

Osteosarcoma (OS) is the most common form of malignant bone tumor found in childhood and adolescence. Although its incidence rate is low among cancers, the prognosis of OS is usually poor. Although some biomarkers, such as p53, have been identified in OS, the association between the biomarkers and clinical outcome is not well understood. Thus, it is necessary to establish a method to identify patients diagnosed with OS at an early stage. It is becoming obvious that anti-tumor-associated antigens (TAAs) autoantibodies (TAAbs) in sera could be used as serological biomarkers in the detection of many different types of cancers. This notion indicates that TAAbs are considered as immunological “sentinels” associated with tumorigenesis underlying molecular events. It provides new insights into the molecular and cellular biology of the differential diagnosis of cancers. What’s more, it is reported that a customized TAA array could significantly increase the sensitivity/specificity. TAA arrays also have great application prospects in detecting cancer at an early stage, monitoring cancer progression, discovering new therapeutic targets, and designing personalized treatment. In this review, we provide an overview of the TAAs identified in OS as well as the possibility that TAAs and TAAbs system be used as biomarkers in the immunodiagnosis and prognosis of OS.

## Introduction

Osteosarcoma (OS) is characterized by the production of bone-like substances by malignant osteoblasts, which is the most common and highly malignant primary bone tumor that originates from primary osteoblasts (1). It is, like all other sarcomas, rare with an incidence rate of less than 1% of all cancers diagnosed (2). It is estimated by the American Cancer Society that approximately 800 new cases arise in the USA each year, and about 400 of them are children and teens. OS is the most common form of bone cancer in children and adolescents ageing from 10 to 20 years old (3). Although the incidence rate of OS is relatively low among all cancers, OS is highly malignant and can often be neglected on misidentification with benign lesions or trauma since the initial symptoms of the disease are commonly quite nonspecific and subtle. Furthermore, OS progresses aggressively. About 20% of the patients have metastases usually detected in the lung when initially diagnosed (4–7). OS is one of the most dangerous primary malignant tumors in childhood and adolescence, resulting in a high rate of amputation, disability, and death. At present, therapy for OS is still inadequate. The five-year survival rate is approximately 60% even after pre- and post-operative, neoadjuvant chemotherapy and excision of operable lesions (8). Moreover, tumor size and metastases presented at initial diagnosis always portend a worse outcome (5, 9). Thus, a critical need in the diagnosis and treatment of OS is to select an optimal array of sera biomarkers that can be used in clinic. These biomarkers could assist to detect tumors at an early stage with high specificity/sensitivity and limited invasiveness. This array could help to predict whether patients in need of treatment will develop aggressive tumors.

Over the last few decades, many studies demonstrated that these autologous cells developing into tumors contained tumor-associated-antigens. The abnormal exposure or presentation of these antigens recognized by the human immune system could further trigger autoantibodies, that have been termed tumor-associated autoantibodies (TAAbs), against these cellular antigens. This notion has come from evidence that TAAbs are immunological “sentinels” associated with tumorigenesis underlying molecular events (10–12). The content of TAAbs could increase in the very early stage during carcinogenesis (13). TAAbs are stable with high levels in patients’ sera even though the level of the corresponding antigens is low (14) or even after removal of these antigens (15, 16). Such benefits of TAAbs have triggered a growing enthusiasm for applying these TAAbs as serological cancer biomarkers. Moreover, in recent years, increasing attention has been given in humoral immunity to TAAs in particular clinical fields, such as the possible use of TAAs and TAAbs systems as cancer biomarkers not only in detecting cancer at an early stage, but also to monitor cancer progression, discover new therapeutic targets, and design personalized therapeutic interventions (17).

At present, emerging studies engaged at the molecular markers or pathways on OS have revealed the key roles of these molecules (13) in OS tumorigenesis and prognosis. These molecular markers could potentially be used to predict the diagnostic accuracy or micrometastasis when diagnosed and when to treat with chemotherapy. Further, such pathways could also be possible targets for new chemotherapeutic agents in OS. The information of the TAAs of human OS is still inadequate. There are too many genes and corresponding protein products, like melanoma-associated antigen (MAGE) (18), HER2 (19), p53 (20), HSP (21), squamous cell carcinoma antigen recognized by T cells (SART1) (22), SART3 (23), or papillomavirus binding factor (PBF) (24), which were reported to be expressed in OS. Unfortunately, it is generally recognized that they are still insufficient in the application of available clinical information in diagnosing cancer early, designing personalized treatment, and predicting prognosis. Therefore, it is necessary to develop innovative diagnostic and prognostic tools to effectively manage OS by utilizing clinical biomarkers.

This review aims to summarize the established and experimental TAAs and TAAbs tested in OS in recent years. It is useful to summarize the idea and possibility of detecting TAAs and TAAbs in the immunodiagnosis and prognosis of OS.

## TAAs in OS

It is now evident that the sera of cancer patients comprise autoantibodies that can react with TAAs. These TAAs are varied and contain a unique group of autologous cellular antigens (10, 25, 26), including the tumor suppressor p53 (27, 28), oncogene products such as HER-2/neu and ras (29), proteins that protect mRNAs from degradation such as p62 (30) and CRD-BP (31), onconeural antigens (32), differentiation-antigens such as tyrosinase and the cancer/testis antigens (33), and anti-apoptotic proteins such as survivin (34) and LEDGF (35). The factors leading to the production of these autoantibodies are not fully understood. The existing studies suggested that many target antigens were cellular proteins, and their abnormal regulation or overexpression may lead to tumorigenesis. This could take p53 for example (27, 28). In the case of mRNA binding protein p62, a fetal protein missing in adult tissues, immunogenicity seems to be associated with abnormal expression of p62 in cells of tumor (36). In some cancer patients, the immune system seems to have the ability to sense these abnormalities and react by producing autoantibodies (37). Autoantibodies associated with a particular type of cancer are targeted at proteins that are abnormally regulated or activated in the molecular pathways involved in the malignant transformation of that particular type of cancer (38). Taken together, TAAbs may be considered as the reporter of abnormal cellular mechanisms during tumorigenesis (10).

Although the reports on types and functions of TAAs in OS are still limited, these tumors might express some diagnostic and/or therapeutic targets. The numerous TAAs summarized in **Table 1** have been described in previous studies. In human OS, the high expression rate of HER2/erbB-2 was 40-45%, which was related to poor prognosis, early lung metastases, and poor response to preoperative chemotherapy (39, 40). p53 was localized in euchromatic areas of nuclei of OS cells, and was involved in the development of OS, but not correlated with any clinical factors (20, 41). Some previous reports had shown that the expression of P-glycoprotein may have an association with the increasing risk of chemotherapy resistance (42, 43, 76, 77). Afterward, it was reported that hsp27 was overexpressed related to the poor prognosis of OS (44). Sudo et al. suggested that melanoma antigen (GAGE) family members were expressed in substantial numbers of OS as tumor-rejection antigens in the main histocompatibility class-I restriction mode (18). And in sarcoma cell lines, hsp72 was selectively expressed on the cell surface, overcoming the protective effect and acting as a target for natural killer cells (46), which was related to the good response of neoadjuvant chemotherapy (47). The GD2 ganglioside was found overexpressed in OS (48) and later, some groups demonstrated that the therapy anti-GD2 antibody can improve the survival rate of high-risk neuroblastoma (49). Two tumor-rejection antigens, SART1 (22) and SART3 (23), were reported to be expressed in OS, which suggests that these proteins and their derived peptides could be used as specific immunotherapy molecules in OS patients with HLA-A24+ or malignant fibrous histiocytosis. It was the uniform expression of B7-H3 (8H9 antigen) on the cell membrane that makes it an attractive candidate for targeted immunotherapy (50). Additionally, a cell adhesion molecule, the human METCAM/MUC18 (melanoma antigen/MUC18), with a high expression level in OS played an important role in the metastasis of OS, suggesting that ABX-MA1 might be a new immunotherapeutic approach for OS (51). Furthermore, the study also concluded that, by using a preclinical model, anti-MUC18 antibodies could inhibit the process of OS metastases (51). In the past few years, a relationship between the expression of CXCR4 and initial metastases was discovered (52). SAA expressed higher in OS than benign bone tumors and normal subjects (53). CLUAP1 as potentially a prognostic/diagnostic marker for OS (54) has been suggested. Jacobs et al. reported that all nine OS tissue samples expressed *GAGE-1, 2*, and *8*, and eight of nine expressed NYESO-1, application of cancer-testis antigens or cancer germline genes expressed in solid tumor research of pediatrics (55). Like the other tumors, survivin was also overexpressed in OS as an anti-apoptotic molecule. Several investigators had revealed that the overexpression of survivin was associated with worse clinical outcome, which may be used as an independent predictor for OS patients in survival field (56). More recently, Maehara et al. showed that the expression level of midkine, a heparin-binding growth factor midkine, has an association with the prognosis of OS patients. Anti-midkine functional antibodies can effectively inhibit the growth of OS cells *in vitro* (57).

**Table 1:** Identification of TAAs or TAAbs analyzed in multiple studies.

TAA or TAAb	Description	Observation in OS	Ref.
HER2	Oncogene	Correlates with poor prognosis for patients with OS.	(39, 40)
P53	Tumor suppressor	Fails as a maker in OS because of no significant correlation between p53 expression and the clinical outcome and response to chemotherapy.	(20, 41)
P-glycoprotein	ATP-binding cassette (ABC) transporters	Increased risk for chemotherapy resistance.	(42–43)
Hsp27	Heat shock protein 27, protein chaperone and antioxidant	Correlates with poor prognosis for patients with OS.	(44)
MAGEA	Melanoma antigen family A	Expressed in substantial numbers of OS in a major histocompatibility class-I-restricted manner.	(18, 45)
Hsp72	Heat shock protein 70 family and a chaperone protein	Correlates with a good response to neoadjuvant chemotherapy.	(46, 47)
GD2	Disialoganglioside GD2, a sialic acid-containing glycosphingolipid	Overexpressed in OS.	(48, 49)
SART1, SART3	Squamous cell carcinoma antigen recognized by T cells, tumor-rejection antigens	Potentially used in specific immunotherapies HLA-A24^+^ patients with OS or malignant fibrous histiocytosis.	(22, 23)
B7-H3	58 kDa glycosylated tumor-associated protein antigen	Potential molecules for use in specific immunotherapies for HLA-A24^+^ patients with OS or malignant fibrous histiocytosis.	(50)
Melanoma antigen MUC18	MCAM (melanoma cell adhesion molecule) and as CD146 (endothelial antigen)	Correlates directly with tumor progression and metastatic potential.	(51)
CXCR4	Chemokine receptor type 4, an alpha-chemokine receptor	Potentially used as a prognostic factor and as a predictor of potential metastatic development in OS.	(52)
SAA	Serum amyloid A, a family of apolipoproteins associated with high-density lipoprotein (HDL)	Increased SAA levels associated with type of tumor and high-risk OS development.	(53)
CLUAP1	Clustering associated protein 1	Potentially used as a prognostic/diagnostic marker and/or for a target of immunotherapy of OS.	(54)
GAGE 1,2,8	g melanoma antigen (GAGE)	Expressed *GAGE-1, 2, 8* in all 9 OS tissue samples.	(55)
NY-ESO-1	New York esophageal squamous cell carcinoma 1 (NY-ESO-1), a cancer-testis antigen	Expressed NY-ESO-1 in 8 of 9 OS tissue samples.	(55)
Survivin	Inhibitor of apoptosis	Potentially used as an independent predictor of survival for OS patients.	(56)
Midkine	Heparin-binding growth factor	Correlates with the prognosis of patients with OS.	(57)
OSAA-3 and OSAA-5	Unknown	Potentially used as candidates for diagnosis and targets for immunotherapy in OS.	(58)
PBF	Papillomavirus binding factor (PBF)	May contribute to peptide-based vaccination and/or adoptive antigen-specific T-cell therapy of patients with OS and other bone and soft tissue tumors.	(59)
TEM1	Tumor endothelial marker 1 (TEM1), prototypical member of a family of genes expressed in the stroma of tumors.	Potentially used as a target protein for selective therapeutic intervention.	(60)
IL-11Rα	Interleukin-11 receptor alpha-chain	May represent a new therapy for patients with OS pulmonary metastases.	(61)
FAP	Fibroblast activation protein	Might be considered as a novel therapeutic target against this cancer.	(62)
Tim-3	T-cell immunoglobulin and mucin domain-3–containing molecule 3	Potential diagnostic and prognostic biomarker for OS progression.	(63)
PCNA	proliferating cell nuclear antigen	Inhibited OS cell proliferation	(64)
CEACAM6	Carcinoembryonic antigen-related cell adhesion molecule 6	A potential therapeutic target for the treatment of metastatic OS	(65)
ANG-IgM	angiogenin	Serve as a biomarker for increased risk of OS	(66)
CDK5	Cyclin-dependent kinase 5	Pro-malignant role	(67)
CEACAM1	Carcinoembryonic antigen related cell adhesion molecule 1	May be a prognostic biomarker for OS	(68)
EZH2	Enhancer of zeste homologue 2	May be tumor-associated antigens of OS	(69)
BMI-1	B cell‐specific Moloney murine leukemia virus integration site 1	May be tumor-associated antigens of OS	(69)
p16INK4A	Cyclin-dependent kinase inhibitor 2A	May be a useful biomarker to guide the treatment of OS.	(70, 71)
PRDX 2	peroxiredoxin 2	A candidate for chemotherapy responsiveness marker in OS	(72)
CXCL4, CXCL6	CXC chemokines	Associated with OS patient outcomes	(73)
Anti-hsp60 antibody	Autoantibodies against heat shock protein60	Increases of anti-hsp60 antibodies at the time of first diagnosis of OS.	(74)
Anti-hsp90 antibody	Autoantibodies against heat shock protein90	Correlates with a good response to neoadjuvant chemotherapy and their absence correlates with the occurrence of metastases.	(75)
Anti-MUC18 antibody	Autoantibody against MUC18	Inhibits the development of OS metastases in a preclinical model.	(51)
Anti-midkine	Autoantibody against midline	Inhibits growth of OS cells *in vitro.*	(57)

In the recent past, several studies have done their best to verify new targeting sites for immunotherapy exploiting neither humoral or cellular immune responses to OS, although their precise role in cell biology remains unclear. Among them, OSAA-3 and OSAA-5, two serological antigens, were identified exclusively in the sera of OS patients, but not in normal individuals, which suggests that these two antigens’ immune responses were related to OS (58). Papillomavirus binding factor (PBF) was identified by derived cDNA library screening with autologous tumor-reactive CD8+T cells. PBF was overexpressed in most OS and might be helpful for peptide-based vaccine inoculation and/or adoptive antigen-specific T-cell therapy in OS patients as well as in other bone and soft tissue tumors (59). The report by Rouleau et al. showed that the measured level of endosialin/tumor endothelial marker 1 (TEM1) was low in normal tissues, but in several sarcoma subtypes it was in high levels, suggesting that TEM1 may work as a suitable target protein for selecting therapeutic intervention (60). In more recent years, researchers found that interleukin 11 receptor alpha (IL11Rα) (61) and fibroblast activation protein (FAP) (62) were selectively expressed in OS patients compared with healthy groups, which suggests that they may play roles during the process of OS development and progression.

On the other hand, the recognition that the production of TAAbs was stimulated by human tumors has opened a new chapter in cancer biology. More and more studies began to focus on the possibility that autoantibodies may be used as serological tools to diagnose and manage cancer early (78). It has been reported that the titer of anti-hsp60 autoantibody was increased in OS patients but it was not found to be associated with clinical parameters (74). One year later, the other group reported that such immunoreactivity against hsp90 may have predictive value in OS patients because the presence of anti-hsp90 autoantibodies was associated with a good response to neoadjuvant chemotherapy, and their absence was associated with the occurrence of metastasis (75).

Upon the current limited information, TAAbs in OS seem to target proteins involved in tumorigenesis, with high expression in bone tumors. Unfortunately, many published studies on the identification of TAAs have failed to further detail this association. Additionally, since molecular diagnostic and/or prognostic markers have not been established clinically, risk stratification is mainly based on the initial stage of the illness and the reaction to chemotherapy. It would be essential to develop a new focus to identify diagnostic and prognostic indicators to detect these drug-resistant tumors as soon as possible so that more aggressive treatment can be used to improve the outcomes at the first stage of diseases.

## Approaches to TAAs Identification

In the past few decades, several approaches have been used to identify TAAs. The most successful methods are serological screening of cDNA expression libraries and phage display libraries, and the latest methods based on proteomics. Based on these techniques, putative TAAs with high-titer reactivity were identified in sera. Subsequently, TAAbs from these sera were used to isolate the antigen DNA sequence from the cDNA expression libraries. In this way, several new TAAs, including p62 (30) and p90 (79), were discovered in our studies. Afterward, several new and previously defined TAAs (25) were identified with cancer sera using a method called serological analysis of recombinant cDNA expression libraries (SEREX), which was an improvement of the method before (80). In cases of OS, it was the SEREX method that could identify new and previously defined TAAs, including CLUAP1 (54), OSAA-3, and OSAA-5 (58).

**Figure 1:**
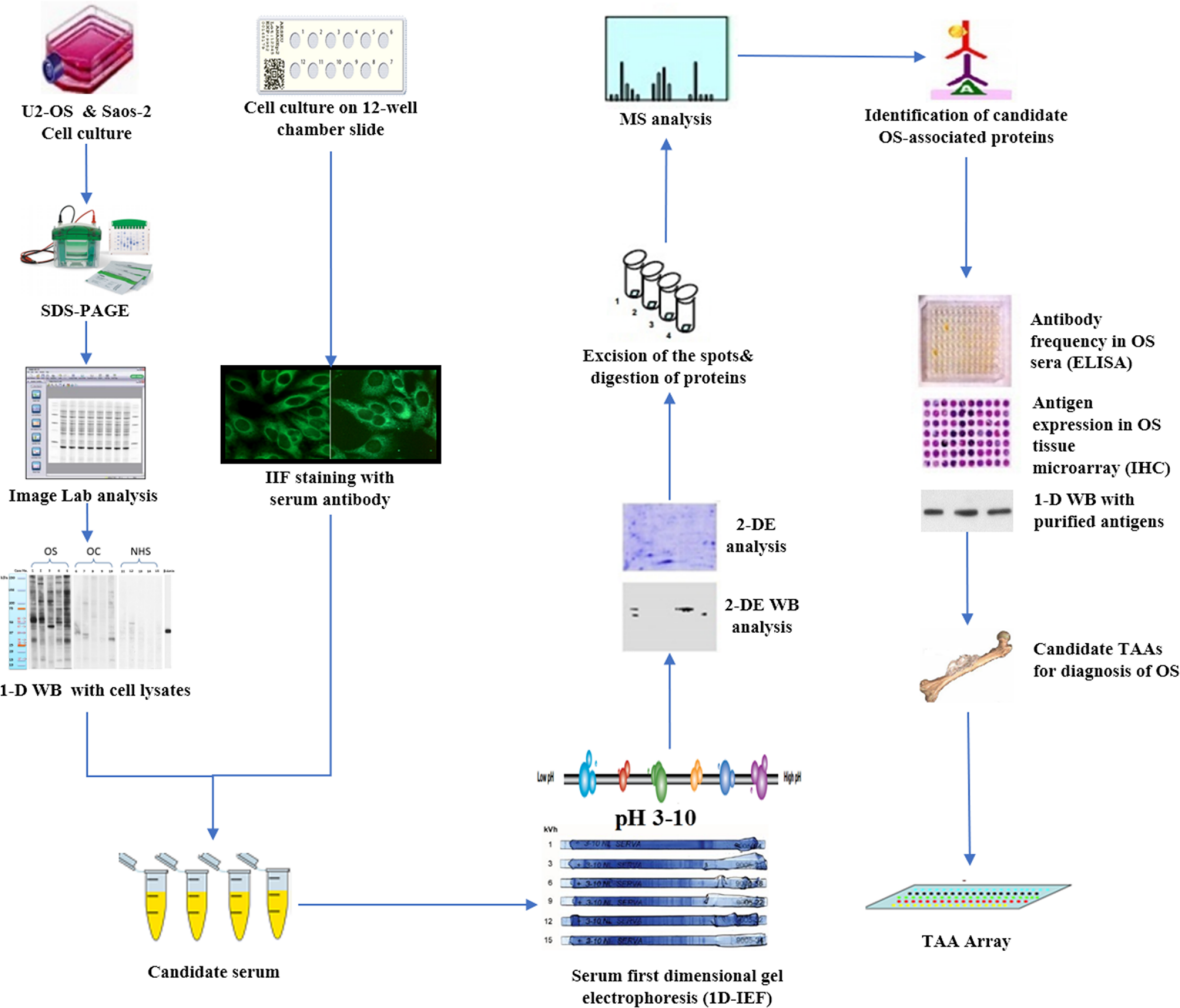
Schematic representation of identification and validation of TAAs using serological proteome analysis (SERPA) approach. In brief, the sera from OS patients and controls were initially examined using extracts of culture cells as a source of antigens in Western blotting and by indirect immunofluorescence (IIF) on whole cells. With these two techniques, we identify sera which have high-titer fluorescent staining or strong signals to cell extracts on Western blotting and narrow the targeting proteins on specific molecular weight bands, and subsequently use the antibodies in these sera as probes in immunoproteomic screening. Cell extracts of cultured human cells was also applied onto the first-dimension gel (isoelectrofocusing gel), and subsequently loaded onto the second-dimension gel (2-DE-SDS-PAGE). The proteins were transferred to the nitrocellulose membrane or visualized by silver staining or Coomassie brilliant blue staining. After immunoblotting with OS sera and control sera, a number of protein spots of interest were excised from the 2-DE gels, digested by trypsin, and subsequently analyzed by mass spectrometry (MS). In subsequent studies, we will characterize the identified cellular proteins that are potential biomarkers in OS.

With the development of proteomic technology in recent years, the field of bone cancer has entered the era of proteomic research, which aims to identify serum biomarkers using proteomics methods, to carry out early and non-invasive diagnosis of cancer, and monitor the progress of the tumor. By using two-dimensional gel electrophoresis (2-DE) system and mass spectroscopy, one method involved direct analysis of human cancer sera to identify specific protein characteristics of different tumor types (6, 81–84). Li et al. used SELDI-TOF-MS to analyze the patients’ sera of OS and osteochondroma, which provided the first example for the identification and verification of the protein biomarker characteristics of OS and found a unique cluster of proteins in the data of patients with bone tumor, which were discrete SAA subtypes (53). A second approach was used in our research (**Figure 1**) and focused on developing a serum autoantibody library for cancer patients to identify TAAs, in order to better diagnose and manage OS by using customized TAA panels or arrays (78, 85). which was termed as serological proteome analysis (SERPA) (86). Compared with SEREX, the technology of the SERPA allows individuals to screen a large number of sera and to identify a large number of candidate TAAs in a shorter time. The proteome-based approach, commonly known as immunoproteomics (80), can also discriminate antigen subtypes and detect the presence of post-translational modified autoantibodies for specific targets. Moreover, this also sheds light on rapid progress in determining vaccine-associated protein antigens (87), immune-related substances, and biomarkers for disease diagnosis and prognosis (80). In recent years, this approach has been used by our lab to extensively screen sera of certain types of cancer patients such as hepatocellular carcinoma (HCC) (88), esophageal squamous cell carcinoma (ESCC) (89), as well as sera from patients with precancerous lesions such as liver fibrosis (90), to identify and characterize the latent TAAs. To support the development and characterization of TAAs in our lab, this protocol was developed to screen the immune sera of patients. As shown in **Figure 1**, Western blotting and indirect immunofluorescence (IIF) were used to detect the sera from OS patients and corresponding healthy controls, initially examined by using proteins extracted from tissue culture cells as antigens source. By using these two techniques, the western blotting method identified sera with high-titer fluorescent staining or react with cell extracts on strong signals, then the antibodies were used in these sera as probes for immunoproteomic screening. After cultured human cell extract was applied onto the isoelectrofocusing gel (first-dimensional gel), it was subsequently loaded onto the (2D-SDS-PAGE). Right after the proteins transferred to the nitrocellulose membrane (NC membrane), silver staining or Coomassie brilliant blue staining was used to visualize the spots. Some protein spots were removed from 2D gels and digested with trypsin after immunoblotting with OS sera and control sera, then liquid chromatography−tandem mass spectrometry (LC−MS/MS) was used to do some analysis. In the following studies, a variety of methods, including Enzyme-Linked Immunosorbent Assay (ELISA), 1D Western blotting, and immunohistochemistry (IHC) with tissue arrays, were used to comprehensively characterize and verify the candidate TAAs and TAAbs systems. In addition to the above methods, single molecule array (Simoa) is a new super sensitive detection technology based on the sandwich method of digital ELISA and combined with high-throughput array technology (91, 92). It has a sensitivity 1000 times higher than ordinary ELISA (93). It also has obvious advantages for the detection of low abundance protein markers, and the detection limit can be as low as fg/ml (94). These screening systems have potential application value in cancer immunodiagnosis. Afterwards, the sensitivity and specificity of different antigen-antibody systems as certain types of cancer markers was evaluated for “TAA arrays” systems developed for diagnosing, predicting cancer, and tracking responses of patients treated in the future.

## Application of TAA-Arrays in OS

Interestingly, TAAbs used as serological markers for the further use of cancer diagnosis stems from the recognition that the expression level of these autoantibodies is usually absent or present in low levels in normal persons and non-cancer patients (85). Comparted with other tumor markers, they present persistence and stability circulation in the sera of cancer patients and may present before the development of cancer, giving them greater early diagnostic and/or prognostic potential (38). Compared with autoimmune diseases, the presence of a particular autoantibody may have diagnostic value; when evaluated individually TAAb has little diagnostic value. This is mainly because of their low frequency, sensitivity, and specificity. Such a limitation has been observed in our previous study and we found it would be optimized by using a properly selected TAA mini-arrays. Furthermore, different TAA arrays were used to diagnose different types of cancer to achieve the required sensitivity and specificity. This then makes immunodiagnosis a feasible auxiliary means to diagnosing tumors and even in predicting prognosis (26).

Our pioneering findings provided evidence that the ability of autoantibodies to detect cancers could be substantially improved by using a series of several TAAs, such as mini-arrays, as target antigens (26, 95, 96). For instance, this mini-array comprised 14 full-length recombinant proteins expressed from cDNAs encoding survivin, CAPERα, RalA, p62, Koc, MDM2, cyclinB1, p53, 14-3-3ζ, p90, IMP1, c-Myc, NPM1, and p16. This mini-array was customized for the detection of hepatocellular carcinoma (HCC). The frequency of autoantibodies to any of these individual TAA was variable, ranging from 5.6% to 21.1% in HCC (97). Nevertheless, with the continuous addition of TAAs in the final 14 TAAs, the positive antibody response of HCC gradually increased to 69.7% in HCC (97). These data indicate that the combined application of multiple TAAs has a higher sensitivity in the serological diagnosis of cancer. More recently, we have evaluated 29 protein antigens identified in OS sera. It was found that only eight protein antigens - DSF70, HMGB1, HCC1, RalA, c-Myc, AnnexinA1, IMP1, and PBP - can induce significantly higher antibody responses in patients with OS with the highest sensitivity and specificity of 0.66 and 0.95, respectively, compared with normal individuals (unpublished data). In a further study, using the panel of these eight TAAs contributes to a 70.7% increase in antibody-positive reactions with an observed AUC of 0.972 (95% CI: 0.867-0.988). These preliminary data extensively supported that not all proteins identified in cancer can be used as be potential TAAs in OS immunodiagnosis or prognosis; only some of them can induce immune responses.

In addition to being potential biomarkers for the immunodiagnosis or prognosis of OS, TAA also has potential as a target for immunotherapy. Vaccinations including peptide-based vaccines are a promising active treatments as more and more TAAs become available for tumor immunotherapy, with the advantage of being easily produced and having minimal toxicity (98–101). For instance, MUC4 could be used as a candidate therapy for the treatment of pancreatic cancer (102). Studies conducted personalized TAA panels to treat patients with glioblastoma multiforme or advanced lung cancer (103). More effective vaccine regimes about TAAs, as well as the mechanism of action of vaccines, should be studied in the future.

## Conclusions

In conclusion, a critical need in the diagnosis and management of OS is to identify a convincing combination of biomarkers used in clinic. These biomarkers could detect tumors at an early stage with limited invasiveness as well as high sensitivity/specificity, which could accurately predict which patients will develop aggressive tumors requiring treatment. Recent research on the TAAs and TAAbs provided a great promise in discovering new tumor biomarkers. The key to these TAAs or TAAbs is to illustrate a new understanding of the molecular mechanisms associated with the skeletal consequences of malignant tumors. There is growing evidence suggesting these tumors may express multiple diagnostic and/or therapeutic targets, although the information on the repertoire and function of TAA in OS is still limited. Our previous studies have provided strong evidence that enhance cancer detection and treatment; TAA arrays provide promising and powerful tools, even though the clinical application of TAA arrays is still in its infancy. It would be necessary to improve the sensitivity and specificity of TAA by identifying new TAAs and systematically defining the optimal combination of TAAs before TAA arrays could be widely used as tools in screening programs for cancer diagnosis or monitoring cancer progression and guiding therapeutic interventions. The results of these studies are greatly anticipated.

## Author Contributions

ZG and WL conceived the study. JL and BQ drafted the application sections; they contributed equally to this work. MH, YM, and DL revised and approved the final manuscript. All authors contributed to the article and approved the submitted version.

## Funding

This work was supported by the National Natural Science Foundation of China (82004397), the Major Project of TCM research in Henan Province (2018ZYZD01 and 20-21ZYZD12), the Major Project of Science and Technology in Henan Province (212102310152), and the Project of Health Guiding Plan in Luoyang (1930005A).

## Conflict of Interest

The authors declare that the research was conducted in the absence of any commercial or financial relationships that could be construed as a potential conflict of interest.

The reviewer JS declared a shared affiliation, with no collaboration, with one of the authors BQ to the handling editor at the time of the review.
